# Nursing as a Functional System of Society. A Systems Theoretical Perspective on Nursing and the Research Object of Nursing Science

**DOI:** 10.1111/nup.70014

**Published:** 2025-01-27

**Authors:** Christopher Dietrich

**Affiliations:** ^1^ State Examination in Vocational Education, Vocational Specialization: Nursing and Health Science; Second Subject: Social Studies. (Germany, Bavaria; comp. Master Degree); Scientific Assistant at Medical School Berlin Berlin Germany; ^2^ Department Medizinpädagogik Medical School Berlin Berlin Germany

**Keywords:** functional differentiation, Luhmann, nursing theory, sociology of nursing, systems theory

## Abstract

The transformation of societies' age structures has intensified the need for nursing care, especially in economically developed regions of the world. This will necessitate societal decisions that determine how care needs are met in the long term. This article offers a sociological perspective on nursing care using Luhmann's systems theory. To make the designation of a functional nursing system with independent observation plausible, social changes were traced based on historical events, semantics, and other social structures to develop the primary view of the nursing system. On this basis, a functional definition of the nursing system and its relationship to problems and problem‐solving is possible. This proposal is intended to clarify the fundamental questions of nursing science: What is nursing and what is behind it? Through abstraction, this article develops a unified representation of nursing's distinct way of observation to support the determination of a unique research object for nursing science as an academic discipline. In line with Brandenburg's statement that nursing science must follow the interests of others as long as it is not possible to conquer a terrain occupied by the discipline independently, the need to develop a genuine discipline remains. Only then, it is assumed, can nursing science significantly contribute to other functional systems and to societal decisions that will determine how care needs are addressed in the future.

## 1 Introduction

1.1

When looking for answers to the question of what nursing^1^ is, these can be divided into two classes: nominalist and essentialist conceptions. Following Popper (1957, 26), nominalist conceptions describe nursing within its main framework and requirements, as proposed in the ANA definition (2010). What is called an essentialist understanding is found in definitions such as “nursing is what nurses do.” (see Cash 1990, 250) While nominalist definitions fail to identify what is not essential to nursing, essentialist definitions equate nurses' actions with nursing. These views reflect the difficulties of the major definitions of nursing in terms of specificity, which leads to the realm of “grand theories” of nursing.

A stop in the development of grand theories of nursing must be noted since the late 1980s. This interruption is due to their lack of applicability, and middle‐ranged theories have received elevated scientific attention (McKenna, Pajnkihar, and Murphy 2014, 65). Critical perspectives on grand theories emphasize that they were designed without consideration of temporal and spatial contexts (Brandenburg 2019, 143). In addition, the normativity^2^ resulting from the subjective attitude of the authors is criticized (Schaeffer and Moers 2011, 55). But focusing on mid‐range theories may improve understanding of nursing in particular situations or of nursing phenomena (McKenna, Pajnkihar, and Murphy 2014, 66), but it hinders reflection on key aspects of nursing, such as the definition of nursing itself. To highlight just two problematic constellations that complicate the conceptualization of nursing, international and setting‐specific differences can be mentioned.

But those aspects only scratches the surface. The missing specification is particularly true for occupations that have been traditionally deemed as the domain of women, as they do not arise from autonomous issues. In the case of nurses, first religious professions and then physicians created their fields of action (Gamarnikow 2013). Both combined tasks that were considered inferior but necessary for their decision‐making process, as was common in women's occupations (Weinbach 2004, 131). In defining nursing, the challenge is to find a theoretical framework that does not focus on the undifferentiated occupational or organizational tasks of nurses. However, if nursing science takes seriously its conceptual position as a practice discipline (McKenna, Pajnkihar, and Murphy 2014, 176), it must take into account existing conditions. But for the contingency^3^ reflection of a practice discipline, there is no other theoretical guideline than its practical reality. If an organization were to succeed in integrating educational or legal advisory roles into the professional expectations of nurses, it can be assumed that nursing scientists would subsequently provide theoretical justification to generate contingency.^4^ 

Rather, it should be clarified what makes nursing action possible in terms of mutual understanding and whether there is a difference in the form of communication compared to others. However, without a clear and well‐founded understanding of nursing, it is difficult to accomplish one of the most important tasks in the environment of any true scientific discipline: the definition of a primary object of research (cf. Krishnan 2009, 9). Without a systematic logic in nursing science, the discipline will not succeed in conquering an area that it could occupy independently, and it will ultimately always have to follow the interests of others. (Brandenburg 2019, 144). To put it polemically: The mere addition of terms such as “nursing” or “nurse” to existing scientific questions does not make nursing science a discipline.

The lack of a specific, nontautological definition of nursing is a central problem for nursing science. In order not to participate in the scientific reproduction of the invisibility of women's work due to its problematic measurement of success, definitions of care should avoid anything that portrays care as all‐encompassing and unspecific. However, if nurse's actions are primarily focused on supporting the success of decision‐makers, it must be assumed that social recognition of nursing will be difficult to achieve. Successes are then most likely to be attributed to those involved in making decisions.

To illustrate the usefulness of a sociological perspective, let's assume an undesirable but not uncommon situation in which a person living with dementia resists care interventions. In general, the following interventions by nurses are expected to be nonviolent and involve the appropriate use of understanding and empathetic communication. The sociological inquiry regarding such circumstances is: Why do nurses not decline assistance when their assistance is not desired? Or more precisely: Why are various other options for action excluded that would otherwise be more likely in contact with strangers (cf. Luhmann 2012, 14‐5)? Answering these questions should lead to an interpretation of how the nursing system operates and how problems are dealt with. And the answer will not be found in personal motives or rational calculations (ibid.).

This theoretical approach does not examine nurses' or care recipients' intentions, but instead uses communication and follow‐up communication for analysis. For this, two questions are essential:

– What is nursing?
– What lies behind nursing?

These questions lead directly to Luhmann's systems theory:

“This theory's answer to the question, what is the case?, is: that which is being observed, including the observation of observers. The theory's answer to the question, what is behind the facts?, is: that which the observation fails to observe. This “unmarked space” (Spencer Brown) results from any distinction made by any observer. […] The operation of observing produces social systems and communication. Social systems are self‐referential systems, forced to observe themselves and other things in the act of communication. Communication simultaneously refers to itself and its object.” (Luhmann and Fuchs 1994, 137)

To explain this statement and the somewhat unusual vocabulary, here is a brief introduction to some building blocks of Niklas Luhmann's sociological systems theory.

## 2 Understanding Systems Theory and Initial References to Nursing Care

In 1997, after almost 30 years, Niklas Luhmann completed his work on a universalist theory of society that would be able to describe all social phenomena and their observers and provide concepts for a sociological analysis Luhmann 1995a, 15). He initially relied on Parsons, but radicalized Parsons’ theory. In contrast to Parsons' assumption of social continuity, he did not focus on stability, but on the adaptability of systems (Luhmann 2012, 23). Instead of focusing on social structures (expectations) like Parsons did in his functional structuralism, Luhmann focused on social functions (problem‐handling). This ties society to the adaptation to its problems, which motivate the creation of structures (Luhmann 2012, 252‐3). And every system defines itself through boundaries that specify which phenomena or objects are included (system) or excluded (environment) (Baraldi et. al 2021, 235‐6).

One of the key figures for a theoretical approach is to understand society as the sum of communication, not people. “Man cannot communicate; not even his brain can communicate; not even his consciousness can communicate. Only communication can communicate.” (Luhmann 2002, 169) This separates human consciousness from social interactions; both are understood as systems, but with different operations. While consciousness systems operate from thought to subsequent thought, social systems operate from communication to subsequent communication. And only communication that generates follow‐up communication is considered successful. Personal motives are not considered to play a significant role in why normally available options are considered unelectable and are not chosen. To illustrate the difference between conscious and social systems, we can return to the example of rejected nursing interventions: Nurses may think terrible thoughts about the person living with dementia, but they communicate in a way that does not reveal these thoughts, which is known as “emotional labor” (Hochschild 1983). It must now be theorized how society succeeds in imputing motives to acting people and implanting them (Nassehi 2008, 170).

A. Part of the answer lies in the steady increase in social complexity that Luhmann explains with his theory of evolution. This will be linked to the history of caring. There are three stages to social evolution: Variation, Selection, and Stabilization (Luhmann 2012, 300ff). Social systems produce numerous variations; some are selected and most are rejected, and some selected variations provide more effective problem‐handling than previously stabilized selections and replace them (Luhmann 2012, 305‐8). Some variations are produced in stock when their time has not yet come, as is assumed for the nursing system. These social evolutions introduced more and more complexity into society, which promoted the development of new forms of social differentiation. Luhmann postulates three main stages and characterizes them by their primary differentiation (Luhmann 2013a, 98). The emphasis on the primary is based on the premise that all social stages exhibit a certain degree of segmental, historical and functional differentiation, but that the primary of the stage is associated with the greatest significance for social expectations.
  In segmentally differentiated society, segments form as social systems. These segments are divided into equivalent units at a low level of development (Luhmann 2005, 136). The dominant social distinction was initially between community and noncommunity. Almost everyone experiences integration into a community, and only severe violations of the rules lead to exclusion (cf. Luhmann 2013a, 18). The low complexity enables the satisfaction of commonly known needs with relatively few techniques and allows for the institutionalization of mutual personal help among tribe members (Luhmann 2005, 137). Care in segments can be seen as a social activity offered by members of the community only to its members. The first evidence of members caring for each other dates back more than a million years (Lordkipanidze et al. 2005). Long before the Neolithic revolution and human settlement (~40,000 years BC), caring for others can be considered one of the oldest social technologies.
  The increasing consolidation of the relationship between give and take in segments increases the acceptance of status differences and leads to stratification (Luhmann 2005, 139). Stratification as the primary differentiation of society is the difference between top and bottom, and social classes are almost exclusively determined by birth, such as in feudalistic Europe or in the Indian caste system (Luhmann 2013a, 13). The increasing complexity of society forces and enables the organization of more and more needs, which also gain additional variety. One of the consequences of this is that mutual awareness of each other falls to a low level. The reaction to this is the construction of moral high gods in societies (Whitehouse et al. 2019, 403).
  To include nursing care in these considerations, it is necessary to take a look at the religious system, which has to deal with newly emerging status differences, poverty and exclusion. Religion is also confronted with a society that is unwilling to help strangers. In Luhmann's understanding, all of religion's communication offerings are aimed at the same solution: giving meaning to contingency (Luhmann 2013b, chap. 4.II). As shown in the theory of evolution, religions differ in the way they deal with problems and in some cases create new variants of problem‐handling. Some variations were selected, others were not. The Abrahamic, monotheistic religions propagate charity or similar concepts and direct their activism towards the excluded (Luhmann 2013b, chap. 8.V). The firm contract between unworthy humanity and the one and only (hopefully) well‐meaning God seems to be the driving force behind these changes: a God who no longer engages in fatty sacrifices, but wants people to act according to the divine rules (Assmann 2010, 44). During the plague wave from 235 to 270 AD, Cyprian described how doctors and others would flee if they became aware of the disease (Dörnemann 2003, 336). In contrast, the Christians, a small sect at the time, selflessly cared for the sick, as they knew that if they died, their death would be equivalent to that of a martyr (Marshall 2008, 597). Christianity forged “kinship‐like networks among perfect strangers based on an ethic of sacrificial love. […] Christian ethics turned the chaos of pestilence into a mission field.” (Harper 2019, 156) This difference is also clearly evident in the case of leprosy: while leprosy colonies emerged in Europe around the fifth century and spread as far as the Byzantine Empire, this invention did not motivate stabilization in China and India, although it was brought there by missionaries (Keil 2007, 841f). The treatment of problems in India turned out to be charitable suicide assistance (Byrne 2008, 364). Monotheistic religions now stylize charitable actions as a moral virtue, with the upper class giving money to the poor to gain access to heaven (Luhmann 2005, 138). With this semantic turn, strangers are at this time addressable for help without being part of the same segment, but they must be in need or sick *and* usually belonging to a lower class.
  The functionally differentiated society has developed various functional systems, all of which view society from their own specific perspective.^5^ The religious system was the first to specify itself in relation to the incipient autopoiesis^6^ of the legal and political system. Poverty is now no longer accepted as a God‐given fate, but seen as an educational motivation to work (Luhmann 2005, 140). Luhmann argues that money as a medium of exchange additionally decreases to possibility of helping others, because on the one hand, there are always others who could help more easily with their greater wealth and on the other hand, there are always others who are even more needy. This means that the willingness to help can no longer be justified by external circumstances (Luhmann 2005, 140). However, with the progressive demystification of society, exclusion is no longer seen as a test of faith (for the excluded and the included) and is increasingly associated with individual rather than structural failure. In modern society, help is strongly tied to programs, not to individual needs, because functional systems tie their helping activities to a comparison of facts and programs and can be generally and reliably stabilized in this form (Luhmann 2005, 144).
  In summary, these social developments of the three phases represent an alternation between specification and universalization. On the one hand, the social dimension (who is addressed) tends to universalization; on the other hand, the factual dimension (what is communicated) tends to specification. Initially specified to the members of a segment, then further on mainly to all members of the lower class and finally to all those in need of care (cf. Luhmann 2013a, 18ff; here: inclusion/exclusion). The shifts in the material dimensions are also a product of the increasing complexity and the addressable persons. Due to the mutual acquaintance in segments and their low complexity, segment members were able to communicate with every segment member about almost everything. Through stratificational differentiation, members of higher classes separate their forms of action from those of lower classes and communication becomes increasingly tied to social class. Functional differentiation requires a higher level of specification than mere assignment to a class, starting to focus on problems, not persons.
B. The next part of the answer relates to social development. The growing population in cities and the high interaction rates made successful communication increasingly problematic. Successful communication in the sense of connectable communication is generally assumed to be unlikely (Luhmann 1990b). For the functionally differentiated society, there are two important effects on the acceptance of communication offers. On the one hand, there are the daily interactions with people, some of whom are strangers, who are approached or turn to others. Employees in a store, students in hospital, musicians on the street, for example. On the other hand, the expansion of communication media, from the development of writing to automated advertising spots, has led to a growing number of attention‐seeking communication offers from strangers, making it unlikely that communication will continue successfully. For this event, society develops success media that reduce the choices in interaction, lower the social standards in interaction with strangers and channel communication, and follow‐up communication into narrower channels. Success media combine conditioning and motivation of interaction for all participants (Luhmann 2012, 121f). Conditioning in the form of learning how to interact in certain organizations such as a court or hospital. Motivation in the form that communication does not break off, even if the ongoing communication may not be based on personal interest. The differentiation of these media also drives systems' differentiation and gives rise to the major social functional systems (Luhmann 2012, 122). For instance, the inclusion of a medical report, the medium of medical communication is set and a conversation can be described as medical.^7^ Most functional systems needed a success medium to catalyze their autopoiesis (Baraldi, Corsi, and Esposito 2021, 231).
C. The theory of communication defines communication as “historically concrete and hence context‐dependent activity and not merely the application of rules of correct speech” (Luhmann 2012, 35) In systems theory, action is understood as attribution to an individual. However, the action itself does not participate in society, but only if it is understood by others as communication. Waving is a social action with the intended meaning of being seen, but it is no communication until the addressee perceives it as such and shows his perception. Communication consists of three selections: information, utterance, and comprehension, all of which are based on respective selections (Luhmann 1995a, 147–150). To reduce complexity, communication theory is illustrated by two individuals. Both are provided with a self‐referential, conscious systems. One system is called *ego*, the other *alter*. *Alter* observes its environment with all senses and selects objects of observation, which transform into information. This information is the basis for communication, the second selection in the communication process, which is based on Alter's image of the person addressed (ego). The utterance must also contain information that *alter* thinks *ego* does not have (selection: information/noninformation). *Ego* understands the utterance within his capabilities but not in terms of *alter's* intentions. *Alter* can observe *ego's* understanding by understanding *ego's* subsequent utterance (Luhmann 1992b, 252–255).

In combining media theory with communication theory, Luhmann distinguishes four possible relations of ego/alter and action/experience, and directs communication from alter toward ego (Luhmann 2012, 201). Money, as a medium of the economic system, enables communication about alter's actions (announced products) and ego's expectation (buying/not buying the announced product). For the nursing system, a similar constellation between ego and alter as love can be assumed. Love as a medium of the intimate system bases its communication on the expectation of the alter that the ego understands its experience and relates current actions to this experience. Thus, the nursing system motivates care dependents to show their experience and nurses to relate their actions. But it creates some problems of nursing communication.

Communication theory assumes that there is no shared reality, that there are two consciousness systems that cannot observe what the other observes and that cannot communicate what they think or experience without following the rules of communication. Intersubjectivity, as emphasized in phenomenology (Merleau‐Ponty, Waldenfels, Schmitz), is explained in terms of subjects. Due to the claimed subject status, there must be intersubjectivity, as no one would claim to be the only subject in the world (Luhmann 2013b, 110). These conclusions are reflected in nursing science in an indirect way, but are nevertheless present (see Paley 2019). Paterson & Zderad argue that one cannot escape the intersubjective “character of care when experiencing the phenomenon, whether as a caregiver or as a patient. Consider, for example, some of the most common caregiving activities, such as feeding and being fed, comforting and being comforted, giving and taking medication.” (Paterson and Zderad 1988, 13). But it is fair to say that feeding and being fed, for example, are quite unfamiliar experiences.

Language and concepts can help to capture a person's experiences, but only with a relatively high margin of error, as they usually mask the profound differences in private life experience (see: Roth 2001, 366). However, words cannot translate the psychological experience into social interaction. Even if words would accurately describe the alter's experience, it cannot be assumed that ego (nurse) would relate the same experiences to the words used by the alter. Without the presence of intersubjectivity, nurses must observe communication and draw conclusions about the other person's feelings from the selections made by the care recipient. They need to understand the difference between information and utterances of the alter and reflect on whether a body movement of an unconscious person is a reaction to an action or spontaneous without communicative intention. What is reflected in the nursing system and in nursing science in the ubiquitous use of the concept of intersubjectivity, leads to the media of success in nursing.

## 3 Medium of Nursing

To focus on the medium of nursing, a look at the specific phenomenon of nursing can help to discover it. Consider the person with dementia who is resistant to nursing interventions. The question then arises as to why successful follow‐up communication by nurses can be expected. What causes nurses to adapt their behavior in such a way that the peculiarity of the situation does not seem absurd to them, but so natural that they continue to offer help despite physical resistance, often even against the will of the person in need of care? First, it is important to remember the religious roots and the opportunities that have been created. The brotherly love and functionally equivalent concepts elevated nursing care to a latent function of the religious system (especially in monotheistic religions) and made it possible to care for strangers. Since success media usually occur before the autopoiesis of systems, it can be assumed that the concept of brotherly love is an interesting candidate for the medium of the nursing system.

To explain this, it must be assumed that the motivation to make one's own self‐care deficits public is low. This is mainly because strangers are not expected to tailor their actions to a person's self‐care deficit ‐ although this does not rule out the possibility that they might do so. The medium of the nursing system must ensure that the disclosure of the “unperson” (Luhmann 1995b) motivates the nurses to professionally align their actions without triggering shame.^8^

The adaptation to alter's experience reveals some parallels with the intimate relationship and its medium (‘love’). There, the ego is forced by the narrow framework to constantly adapt its actions to the experience of the alter (Luhmann 2021, 22‐3). With each adaptation the integration of the entire personality of the ego increases at the same time and leads to further expectations of ego, because the ego is “extending the boundaries of the system” (Luhmann 1995a, 195). In this way, the ego confirms the egocentric worldview of the alter, which also means that the ego is expected to confirm the behavior of the alter, which cannot be represented elsewhere (Luhmann 2021, 21).

Nursing theories reflect these mechanisms of love in nursing, as they describe that nurses should “look at and into the inner‐life world through the other person's eyes” (Watson 2008, 194) and tie their measurement of success in meeting the patient's needs “as perceived by the patient” (Meleis 2012, 71). Just as love manifests by allowing the other person to be who they really are (Luhmann 2021, 29), this “giving” is also evident in the semantic of nursing. Therefore, nursing communication is closely linked to the concepts of empathy and intersubjectivity. Empathy, because it includes the attributes of understanding, feeling, and sharing another's feelings while maintaining the distinction between self and the other (Håkansson and Summer Meranius 2021, 306), and intersubjectivity to assert a common shared reality. Attributed empathy can already increase the likelihood of communicating relevant aspects, as an appropriate response to the alter's experience can now be expected. Intersubjectivity also aims at a common reality, which is also useful in reducing social uncertainties. But it must be doubted that these perceptions are more than brain activities of separate consciousness systems that feign this commonality, at least from the perspective of constructivist neuroscience (Roth 2001; Damasio 2010).

However, if the possibility of a change of perspective were to be negated or its limits clarified, many intuitively implemented nursing measures would lose their mutual legitimacy and lead to greater uncertainty of action. The insistence on intersubjectivity, on access to others, even if “we cannot observe what they think, and only we ourselves can observe what we think” (Damasio 2010, 5), fulfills social functions and reduces the complexity of problem‐handling. If nurses are perceived as empathetic, approachable and understanding, it is easier for people in need of care to report unpleasant circumstances (their “unperson”). On the other hand, these attributions usually create trust in the role of the nurses, which is used to facilitate interaction. The fact that the option of brotherly love was chosen as the medium, and not charity or altruism, results on the one hand from the conceptual proximity to love. On the other hand, charity is too strongly directed towards specific individuals (Hamburger 1985, 123‐4) and altruism seems to be an socially unreliable intention. Due to its obligatory character, brotherly love as a medium limits the possibility of rejection of communication more clearly and universalizes the social address.

This tied motivation and conditioning of nursing communication and was suitable as a preadaptive advance for later problem‐handling. It also has a lot to offer in terms of understanding nursing communication: Brotherly love makes it possible to articulate far‐reaching needs without having to respond to the needs of the other person (nurse); it loses its credibility if the actions are superficially only financially motivated; it is perceived as a female rather than a male virtue, and it promotes the revealing of the “unperson.”

## 4 Codification of Nursing

**Figure 1:**
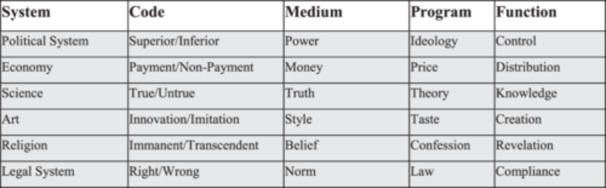
Exemplary selection of functional systems according to Roth (2015, 42).

For functional systems, a binary codification motivates specified selections (Figure 1), generalizable in a system/environment distinction. The modern functionally differentiated society works on society's problems by specifying views on a phenomenon. Every functional system's observation has a specific binary code, and only the positive side of the codification leads to follow‐up communication. Most social phenomena can be separated into different kinds of observations. For example, the aging society causes problems at least in the following systems: political (change in social legislation), medical (increasing complexity of disease patterns), legal (dignified dying), economic (customized products), and nursing (professional care) ‐ provide specified and self‐referential problem handling.

The autopoiesis of the nursing system can be reflected in at least three conceptual semantic shifts: first, especially in monotheistic religions through the development of brotherly love or the like. As Whitehouse et al. point out, the high complexity of societies in most cases precedes the development of moralizing high gods (2023, 135), which marks their existence as a form of problem‐handling. These high gods had the social advantage of animating social cohesion under the guise of benevolent transcendent rule (Whitehouse et al. 2019, 403). The second semantic shift is the introduction of the distinction between religious and secular care. In this context, there is a stronger link between nursing and medicine, which acted as advocate and judge of the legality of women's professional activities on behalf of patriarchal bourgeois society at the end of the 19th century. The third shift is the emphasis on holistic care as distinct from medical observation (Kim 2006, 89). Semantic developments such as these signal processes of idea development without characterizing them as causal. These semantics provide clues as to how concepts contribute to the emergence of new social expectations by increasing the likelihood of certain selections (Luhmann 1993a, 8, 23–26).

Without motivating the autopoiesis of the nursing system, brotherly love as semantic evolution initially motivates people in monotheistic regions to help strangers in need. Following Luhmann's remarks on the distinctions of religious vocations (sin/mercy and suffering/salvation in relation to transcendence; Luhmann 2004, 194), the initial structures of the nursing system are primarily based on the second. The increasing scientification of medicine has supported the autopoiesis of the medical system by excluding religious arguments from diagnostic processes.^9^ In France and Germany, physicians began to complain about Christian nurses who refused to subordinate themselves to the physicians. “Doctors hoped to finally gain recognition of their claim to leadership if nursing was organized in a secular and professional manner” (Kreutzer 2019, 116). Surgeons in the United Kingdom also called for better training after noticing improvements (Dingwall et al. 2002, 71‐2). This can be considered an advocate role, while monitoring educational efforts and their medical utility assigns physicians a judge role (*cf.* Dingwall et al. 2002, 33‐4).

Florence Nightingale and her influence on modern nursing made secular nursing a professional activity (Selanders 2010). The influence of the medical system promoted the development of nursing as a profession—with the caveat that nursing is first and foremost a performance for the medical system. Furthermore, the medical system stimulated changes in the dualism of suffering/salvation into immanent terms, which then shaped nurses' reflection in the background. However, this could only be completed through the following semantic turn.

The overwhelming influence of medical communication and medical observation has led nurses to question their heritage and distance themselves from medicine. In 1970, a new nursing semantics was born from Rogers that linked all nurses' tasks together: holism (Meleis 2012, 13). With this semantic legitimation, any organizational task that was not tied to higher professions could now be the task of a nurse, as it allowed for a consideration of the “whole person” rather than the illness fixation of physicians. Instead of limiting itself to certain factual dimensions, holism claims to be able to consider the whole person.

This holistic approach can also be found in other areas: holistic medicine, holistic life coaching and even holistic real estate agents, for example. Everything seems to have a holistic counterpart, but surprisingly in its functional differentiation. Holistic care is also a semantic that does not adequately describe the social reality of care, not least because holism defies precise definition (Stemmer 1999). The implications of this nursing semantic of nursing can hardly be overestimated. This semantic not only creates contingency for the undifferentiated activities of nurses but also generates structures for the later ANA definition of nursing, which can hardly define what does not belong to nursing. It is challenging to think of another word that could describe the invisible work of nurses more aptly and appreciatively than the veiling concept of holism. Holism forms the bracket that summarizes the undifferentiated activities and remnants that other professions leave behind, and sells them as an independent value. Although the term holism is conceptually inadequate, it is not entirely misleading in describing the organizational tasks of the nurse.

To propose a codification of the nursing system that reflects the religious background and transitions guided by the medical system, the positive side of the observation of the religious profession (“suffering”) is used. The medical influence that socially removed transcendental explanations for illness also motivates the nursing observation to remove religious reflection on the negative side of codification, leading to *non*‐*suffering*. This does not exclude the possibility that the suffering is based on psychological problems, but nursing communication is not aimed at the afterlife to monitor success, but at the current perception of the suffering person.

Suffering comes etymologically from the words *sub* and *bear*, which indicate that something is unbearable for someone (Hoad 2003, 471). This includes passivity and a person's inability to meet their own needs, similar to Orem's self‐care deficit theory (Fawcett 2005, 229). In order to set limits to the nursing system, what can be labeled as suffering is predetermined primarily by religious traditions, such as illness, loneliness, insecurity, pain, injustice, exhaustion, and fear.^10^ Specific forms of suffering can be found in almost all religions. However, not all types of suffering belong to the factual dimension of the nursing system. For example, suffering from wealth ‐ because there are no more desires that can be bought ‐ is excluded from the observation of the nursing system. Some conditions are also perceived as suffering, but are not classified as requiring intervention by the nursing system (e.g. childlessness or divorce), unless the person concerned is dependent on nursing care in other areas. If this is not the case, society expects those affected to manage on their own (with the assistance of friends or family) or to seek professional help. This minimum level for offering communication does not only apply to the nursing system. There is also a minimum level of necessary intervention in other systems. For example, the legal system would not intervene if a child stole another child's lollipop, even though stealing is illegal.

Media and codification answer the two questions “what is nursing” and “what lies behind it,” but cannot provide sufficient information about the function of the nursing system. To make its function, the nursing system must first be distinguished from other systems.

## 5 Differentiation of Systems

As the functionally differentiated society states, communication is less dependent on who is talking than what is talked about, what is the problem which is worked on. For this, functional systems transfer social phenomena into a paradox (problems) and provide self‐referential treatment (function). This does not mean that any word being said in an interaction system belongs to a specific functional system; rather, the point is what communication is guided by, what problem is being focused, and does it need a start from a higher level of complexity (Luhmann 2013b, 9). Thus, some of the systems in direct concurrency limit their communication because the problem handling of other systems is more appropriate. These limitations are not to be understood as a qualitative assessment, but mean that the reasons for problem‐handling are reduced by other systems. For example, successful cooperation between professional and family caregivers may reduce the reasons for the nursing system to initiate communication with the care recipient.

If the proposed codification is correct, the nursing system will begin to address problems when suffering is observed. Care recipients expect caregivers to recognize their negative experiences and choose their actions in relation to these experiences. Positive experiences do not motivate care communication unless they can be associated with negative experiences. This is similar to the way many systems work. In the education system, for example, poor performance is the reason for educational communication, whereas adequate performance is unlikely to lead to educational communication—unless it is related to poor performance.

For many functional systems, the suffering of the individual can serve as a catalyst to initiate the treatment of their problems. However, most systems are not expected to treat the suffering itself. If a functional system can observe suffering, it is considered a symptom of a certain deficit (problem). The communication offered by the system now aims to treat this deficit (function). In psychotherapy, for example, suffering can be productive and unproductive in terms of the suffering person's motivation to change something in their life (Davies 2012, 16ff). However, the problem treatments offered differ, while psychotherapy strengthens self‐efficacy and individual problem‐handling, nursing communication targets the symptoms (alter's experience), and suffering can be found in physical, psychological, and social domains.

Suffering may be the reason for a visit to the doctor, but medical communication uses the utterances of the patient's suffering to make a diagnosis. Attempting to find a cause in an abnormality of bodily homeostasis, or when no diagnosis seems appropriate, medical communication comes to an end. The medical system distinguishes between ill/healthy in its consideration (Luhmann 1990d), and (socially constructed) illness is a necessity for medical communication to continue. Medical communication centers on the human body, and even within psychiatric institutions, medical observation focuses on neurotransmitter dysfunction and pharmaceutical correction options.

In both cases, suffering is not the (main) criterion for success. Because of the passivity of suffering, nursing care must act on the body in order to influence the perception of the conscious systems (e.g. staying with the patient). As a latent function, the nursing system can exert a positive influence on independence and illness through its interventions, just as the medical or healthcare system can reduce suffering through its latent functions. However, maintaining independence or restoring health is not the main part of the nursing system's observation.

The most important differentiation for the functional nursing system is between professionals and family caregivers, as the importance of nursing increases in a care‐dependent and multimorbid society. Caring for family members is not a fundamentally new task for families, but due to the ageing and multimorbid society and the increasing complexity of daily life, families have more problems to deal with (Schulz et al. 2020, 636). The traditionally claimed loss of family function does not imply a decrease in the family's social importance, but a specification with relief on the one hand and intensification on the other (Parsons and Bales 1956, 9–10). Luhmann's perspective on family systems emphasizes their function of perceiving human beings as whole persons^11^ (Luhmann 1990c, 208)—as the holistic view asserts. In a functionally differentiated society, only the family (Baraldi, Corsi, and Esposito 2021, 239) and intimate relationships can fulfill this function for individuals.

The factual dimension of the family system supports the inclusion of all concerns of the family members, and the nonresponse to these concerns (“Why are you late?”) must be credibly and comprehensively justified (Luhmann 1990c, 201–202). The factual dimension of families enables different perceptions of financial issues, health status, housing situation, personal boundaries, etc. and creates the possibility of conflicts between family caregivers and those in need of care (cf. Schulz et al. 2020, 642). Thus, family communication can include the communication of different functional systems on a residual level (Luhmann 1990c, 207), but only for its core members. The most important difference to emphasize is not to say that family caregivers do a poor job because of their unprofessional actions, lack of skills, or lack of knowledge. Disparaging views of family caregivers would negate any success and make their successful efforts seem purely accidental. However, some situations show that care provided by family members is a better choice for the care‐dependent.

The difference between nursing and caring is that all persons can care, but nursing is expected to be given to all—if they are suffering. Thus, nursing as a functional system overcomes the specific person.

## 6 Function of Nursing

If an autopoiesis of nursing as a functioning system is accepted, its function must be defined. Luhmann proposes three duals to observe the function of a functional system:

a. Contingency/necessity
b. Cause/effect
c. Problem/problem‐handling (Luhmann 1970)

A social phenomenon can be described as contingent if more than one solution is considered possible in society. As a result, a problem loses its necessary solution and becomes ambiguous. As stated before, the family system has its specific functions that will be extended by changes in the age structure of society. Families not only have to socialize their children and stabilize the adults' personalities (Parsons and Bales 1956, 16), but maybe they also have to assist their older family members in the progress of “desocializing” from society.^12^

In order to transform an individual problem into a social problem, the existing systems must have inadequate problem‐handling strategies and there must be a higher probability that a large number of people in a society will be confronted with this problem. The nursing system must be seen as a product of increasing long‐term care dependency that is a product of medical treatment successes (Grasekamp 2017). Today's medical system has thus not only favored the autopoiesis of the nursing system as a secondary functional system, but is also confronted with its reflective deficit due to the emergence of the nursing system. The increasing efforts for medical improvements lead to paradoxes in the functional system itself and society because they are too successful. Because of the differentiation of the medical system and its focus on programs^13^ to restore human bodies, problems are not solved. Successful therapies are not an equation to healing, they are just the success of therapy. By recovering the human body, the medical system leaves a body behind which may have been restored though, but this body must expect new and even worse illnesses (Grasekamp 2017, 317). The paradox of the medical system is that by curing an individual, society becomes sicker. With the current state of medicine, society can expect years of reduced quality of life for all family caregivers (Cheng et al. 2022). In some family systems, caregiving is accounted for, in others it is not, but it is simply not reasonable for society to expect that all families will bear the burden of caregiving for many years to come.

This leads to the dualism of problem and problem‐solving. The autopoiesis of the nursing system must be seen in the shown context. The problem must therefore be considered a product of the progress of the economic system in general and the medical system in particular in an ageing society that is increasingly dependent on care by nonfamily members. Suffering, understood as the impossibility of satisfying one's own needs, as a socially mediated condition that is no longer considered bearable, forms the specified problem of the nursing system. The nursing system focuses directly on the negative experiences of the dependent person. Positive changes to these experiences are considered successes in dealing with the problem.

Regarding Luhmann's attribution of helping as the satisfaction of needs under the problem of balancing needs and time (Luhmann 2005, 134), it can be assumed that the function of the nursing system is to substitute the demands of the family system in the broad field of anticipated suffering. But only there: genuine love from relatives, for example, cannot be replaced by professionals. Nowadays, the possibility of replacing family caregivers with external professionals is largely detached from the (moral) coercive nature of family ties. Due to this functional attribution, the autopoiesis of the nursing system cannot be dated back a century or more. Only if the need for care is a social issue, is it plausible to speak of a completed autopoiesis of the nursing system from a functionalist perspective.

## 7 Transfer

The proposed systems theoretical analysis of the nursing system can be integrated into various existing nursing theories. More interestingly, it can motivate a reevaluation of Fawcett's metaparadigm (Fawcett 2005). Its major concepts, environment, person, health, and nursing, have been criticized because nursing emerges from the relationships of the other three concepts and defining nursing as a component of the metaparadigm seems inappropriate (Greb 1997). Instead, nursing theories could now reconsider person, health, and environment in terms of reducing suffering. This does not negate the importance of medical and health knowledge for nurses' actions, but it does provide the main framework for nursing observations. The nursing diagnoses of NANDA (2020) can also be concretized, which can be shown exemplified in case of dementia and its symptom of Chronic confusion. If the diagnosis determines what a person with dementia is suffering from, you know how to provide care.

In relation to Table 1, all three observations show treatment options, all of which can lead to nurse's actions. In this context, the nursing system observes various forms of suffering, such as fear, aggression, passivity, malnutrition or insomnia. For example, aggressive behavior reflects anxiety due to disorientation (suffering), and can be reduced by patient explanations, positioning of familiar objects, etc. This can be useful in defining the object of research in nursing science, which seems to be primarily a science about nurses rather than nursing. Suffering precisely reflects the professional challenges in nursing practice. And it opens the door to a professionalism in dealing with people that cannot be structured by organizational standards (cf. Evetts 2011, 412), but must justify decisions and outcomes in terms of reducing/avoiding suffering.

**Table 1:** Simplified overview of different perspectives on a specific phenomenon.

Chronic Confusion	Medical	Health	Nursing
Observed as	Illness	Difficulty initiating tasks and activities	Suffering
Problem‐solving	Medication	Reality orientation program	Reducing anxiety through patient explanation

## 8 Conclusion

Summarizing the sociological analysis, whose reference points are primarily the social expectations of an interaction, both semantic framings and historical backgrounds are revealed, which have an effect on mutual expectations in nursing interactions. While these expectations are not to be understood as completely deterministic, it may be difficult to circumvent them in the normal course of unreflective behavior. Then certain forms of communication (and their motivated selection) operate in and through the people involved.

If the nurse violates the presumed wishes of a person with dementia, it is only justified if the nurse observes actual or potential suffering. It is then expected that a nurse will take action, and even assertive behavior is tolerated to a certain extent. However, this is expected and justified under the circumstances that the options chosen likely serve to lessen the suffering experienced by the person in need of care.

While such behavior may be tacitly tolerated or even rejected within the nursing system, and only few nurses are likely to openly display such behavior in public, it is the social mechanisms of the nursing system that provoke such behavior. It is therefore not a qualitative assessment or a definition of ‘good care,’ but a description of how and why nursing communication is initiated, carried out and maintained. Of course, the situation described can also be made more positive and the cooperation and participation of those in need of care can be increased through empathic care. However, this only shows a different handling of the problem, not a different problem.

This systems‐theoretical analysis enables a perspective on an independent field of nursing science, which frees it from the impact of holism and can establish its autonomous position. It is based on existing societal expectations for nursing communication, and the analysis presented is merely a theoretical proposal for finding unity in diversity through the lens of abstraction. There is neither a definition of good practice nor information about the necessary qualifications of nursing staff. This cannot and should not be achieved from a systems‐theoretical perspective.

Finally, three suspected or widespread misunderstandings of a functionalist perspective in general and of nursing in particular should be avoided. A sociology of nursing, even if it argues in a functionalist way as here, is not a guide to functionalist care—quite the opposite. The theoretical exclusion of humans from society on the basis of their methodology is not intended to provide a theoretical justification for inhumane treatment, but rather to give nursing science a basis for determining which research priorities arise from the social context of nursing. Second, this description is not intended to reinforce the status quo, but to provide a basis for describing what nursing means in today's society. Therefore, no “what to do now” should follow. Rather, prognostic statements about society should be deliberately concealed due to their high probability of error (Luhmann 1998, 76‐8). The conclusions that can be drawn from this suggestion may become apparent in possible future presents. But this proposal makes it possible to remedy a central theoretical deficit in nursing science. Third, there are arguments in favor of an overly centrist representation of Western ideas. One argument is that the nursing theories received worldwide are mostly of US origin and thus export theoretical understandings of nursing worldwide (Herdman 2001, 5). Additionally, the global trend seems to be that care provided by family members is increasingly losing predictability. It can be assumed that professional nursing care will also be used as a substitute for family in other regions. However, if the assumption of secularized nursing care is correct, there is little to prevent secular brotherly love from stabilizing as a medium of success in these regions as well.

Regardless of the reception of these findings, work on the definition of nursing and the object of research in nursing science should not be abandoned. Their contribution to the governance and planning of nursing in society can hardly be underestimated. The question of a definition of nursing for the 21st century will be answered in view of a growing number of care dependents. It is certainly better if nursing science answers this question as soon as possible.

## Conflicts of Interest

The author declares no conflicts of interest.

## Data Availability

The authors have nothing to report.

## Endnotes

1 The difference between care and nursing is not easy to define. Some theories speak of caring as the core of nursing (Leininger 1977; Schwerin 2004), and Watson equates caring with nursing (Watson 2008, 15). However, following Boykin and Schoenhofer (2001, 1), according to whom everyone is a caregiver, the author prefers to distinguish between caring and nursing in the sense that nursing is a vocational activity and that care can be provided by everyone, but not for everyone. For this reason, the author speaks of a nursing system and not a care system.

2 This can be assumed to be a historically evolved problem. Since the beginnings of nursing research by Nightingale, nursing science has been seen as the key for nurses to become independent, to be perceived as a profession, and to make nursing a recognized women's work (Alligood 2014, 3f). Nursing science has been and continues to be used primarily as a prop for nursing work to ground political and professional claims, rather than to build a critical perspective on its field.

3 Contingency denotes the exclusion of both necessity and impossibility. The word contingency describes a situation in which what is currently true (i.e., not impossible) could also be different (Baraldi et al. 2021, 75).

^4^
Contingency describes the expansion of action and interpretation possibilities: What initially appears as not belonging or impossible becomes integrated as a new option into the potential space.

5 For instance: Ongoing secularization is a religious observation.

6 Autopoiesis is the term borrowed from biology for the self‐generated development and maintenance of social systems (Luhmann 1990a).

7 It is completely irrelevant whether the medical finding is correct, because this finding is the basis for further communication and is therefore a social reality, regardless of its true content.

8 The concept of unperson can be understood as a person's existing but not socially approachable characteristics, comparable to Goffman (1959, 112‐7). Luhmann gives the example of the tendency to epileptic seizures, which, when they become visible, are inevitably attributed to the person (Luhmann 1995b, 149).

9 Luhmann argues that the religious system must deal with increasing social complexity and answer more and more questions, especially those it has not asked itself (Luhmann 1993b, 264).

10 These can also be found as nursing phenomena in the current literature. However, it should be critically noted that the similarity between religious motives of suffering and nursing phenomena does not verify the thesis of a clear connection.

11 But not as whole human beings, but as a fully integrated social address.

12 Sentences such as “I promised my parents I would never put them in a nursing home” are a product of social structural change and would not have been said two hundred years ago for various reasons. Such formulations thus provide access to the new expectation for families to integrate caring for older family members.

13 Programs help functional systems to define the side of the binary code, for the medical system there is diagnostics, for nursing system it is assumed in empathy and/or standardization.
